# Efficacy of bibliotherapy combined with games to reduce fear of the dark in young children: results from a clinical trial

**DOI:** 10.1007/s00431-026-06918-2

**Published:** 2026-04-20

**Authors:** Mireia Orgilés, Àngela Belzunegui-Pastor, Víctor Amorós-Reche, Jose P. Espada

**Affiliations:** Child and Adolescence Research Center, AITANA Research Group, Miguel Hernández University, Avda. de la Universidad, s/n. 03202 Elche (Alicante), Spain

**Keywords:** Nighttime fears, Bibliotherapy, Psychological treatment, Clinical trial

## Abstract

Intense fear of the dark is a common issue among children, which can interfere with their daily functioning at family, social, and academic levels. This study aims to analyze the effectiveness of a psychotherapeutic intervention based on bibliotherapy combined with play to overcome the fear of the dark in children between 4 and 8 years old. A total of 38 children participated, who were assigned to the experimental and control conditions on the waiting list. The bibliotherapy intervention in the experimental condition involved reading a book and playing the games proposed in each chapter. The intervention lasted 4 to 5 weeks. The results indicate a decrease in fears related to nighttime characteristics and imaginary stimuli in the experimental group, whereas no improvements were observed in the control group. Additionally, significant improvements were found in the nighttime behavior of children in the experimental group and their ability to act in dark situations.

*Conclusion*: The findings demonstrate that play-based bibliotherapy is an effective treatment for overcoming children’s fear of the dark. Furthermore, the relevant role of parents in addressing this type of problem is demonstrated.

*Trial registration*: registered on https://ClinicalTrials.gov (ID: NCT07067320). Date of registration: 16/07/2025, retrospectively registered.

**Table:** 

**What is Known:** • *Fear of the dark is common in children and can negatively affect sleep, behavior, and daily functioning.* • *Bibliotherapy is a promising, low-cost intervention for addressing children’s nighttime fears.*
**What is New:** • *Provides evidence for the effectiveness of a home-based bibliotherapy program combined with games for children aged 4–8 in reducing fears and improving nighttime behavior, highlighting the key role of parents.* • *Evaluates an expanded version of the intervention with new components (e.g., gradual exposure, nightmare management). *

**What is Known:**

• *Fear of the dark is common in children and can negatively affect sleep, behavior, and daily functioning.*

• *Bibliotherapy is a promising, low-cost intervention for addressing children’s nighttime fears.*

**What is New:**

• *Provides evidence for the effectiveness of a home-based bibliotherapy program combined with games for children aged 4–8 in reducing fears and improving nighttime behavior, highlighting the key role of parents.*

• *Evaluates an expanded version of the intervention with new components (e.g., gradual exposure, nightmare management). *

Intense fear of the dark is a common problem among children [23, 25]. Fear of the dark can manifest itself through various nighttime fear patterns and coping styles, including low fear and low self-control, high fear and maladaptive coping, moderate fear and adaptive coping, and high fear with maladaptive coping. The most frequent pattern is moderate fear combined with adaptive coping [11]. Nighttime fears are often presented as the main reason for consultation, although they frequently occur as part of a broader anxiety disorder, such as specific phobia or separation anxiety [9].

Studies involving families in the assessment of children’s fears have found that children with fear of the dark often exhibit other associated problems [10]. On one hand, emotional difficulties are common in these children, often manifested through avoidance behaviors at bedtime, such as crying and complaining. On the other hand, behavioral issues are also frequent, including waking up at night to seek attention from parents or siblings or sharing a bed with them. Academic [20] and social development [25] are also negatively affected by fear of the dark, as children with poor sleep quality struggle with concentration and tend to avoid spending the night away from home. Finally, the family environment is significantly impacted, as children with this issue frequently resist going to bed or seek the company of family members during the night [23].

The treatment of choice for fear of the dark is cognitive-behavioral therapy (CBT; [3]). In a clinical trial, Kahn et al. [14] compared the effectiveness of CBT combined with play and parental involvement (CBT-PIP) versus triadic expressive play therapy (TEPT). Their findings indicated that although nocturnal fears significantly decreased in both treatment groups, parents reported more positive outcomes for CBT-PIP, including greater reductions in sleep problems and co-sleeping, as well as higher satisfaction with the intervention. Similarly, Pincus et al. [27] compared the effectiveness of CBT against a home-monitoring control group, finding that only the significant reductions in the CBT group were maintained over time. Cognitive-behavioral therapy with substantial parental involvement has also been found to be effective [8]. Various studies have demonstrated the efficacy of brief and low-cost treatments for reducing nocturnal fears [4, 14, 16]. Among these treatments, bibliotherapy-based interventions have shown promising results [17, 29].

Bibliotherapy-based treatments offer multiple advantages. According to Gordon et al. [13], incorporating story characters as coping models provides a positive way to teach children how to handle dark environments. Additionally, these interventions often integrate effective behavioral strategies, such as counterconditioning techniques, gradual exposure, and modelling. Furthermore, bibliotherapy is a suitable treatment for families who face difficulties attending in-person weekly psychological therapy sessions [28]. It is an easily administered therapeutic option with the potential to enhance motivation for change and incorporate therapeutic components in a format that is engaging for children [7].

Several studies have previously demonstrated the effectiveness of the book *Uncle Lightfoot, Flip that Switch* [5], a bibliotherapy-based treatment for reducing children’s nocturnal fears [15, 17, 21, 30]. This intervention has been found effective in decreasing night-related fears and fear of the dark [15, 17, 30], as well as in improving adaptive nighttime behaviors [15].

This intervention is designed for children aged 3 to 8 years, lasts 4 to 5 weeks, and is implemented by parents with their children at home. It includes in vivo gradual exposure, play, relaxation, verbal prompting, extinction, material and social reinforcement, symbolic modelling, cognitive modification, and parent training [30]. In the study by Santacruz et al. [30], an earlier version of the program was used, consisting of 12 chapters and 9 games. However, the version examined in the present study has been significantly expanded, now comprising 22 chapters and 18 games. While preserving the essential components of the original program, the updated version incorporates elements specifically designed to address difficulties associated with nocturnal fears and maladaptive nighttime behaviors.

Among the new chapters and games, the intervention includes gradual exposure to darkness in everyday situations, such as sleeping alone in one’s own bed, walking in the dark to the bathroom or kitchen, and remaining in bed with the lights off for several minutes. Additionally, activities focused on managing nightmares have been integrated, helping children develop adaptive coping strategies. These enhancements aim to increase the effectiveness of the intervention by promoting a greater generalization of treatment gains.

The aim of the present study was to analyze the effectiveness of a bibliotherapy intervention based on the book *Uncle Lightfoot, Flip that Switch* to reduce fear of the dark and improve nighttime functioning in a sample of Spanish children aged 4 to 8 years. It is expected that, after the bibliotherapy intervention, the children’s nighttime fears will significantly decrease and there will be significant improvements in their nighttime behavior.

## Method

### Participants

The participants were 39 children with nighttime fears, aged between 4 and 8 years, with a mean age of 6.05 (*SD* = 1.337), and 46.2% were girls. None of the children participating in the study was receiving psychological treatment for fear of the dark. The reasons for seeking therapy outside of the program included sadness and/or low self-esteem, behavioral problems, anxiety problems, and autism spectrum disorder. Table 1 presents the sociodemographic variables of the children and their parents of the two experimental conditions.

**Table 1:** Sociodemographic variables of the participants of the experimental and control groups

	Experimental group (*n* = 23)	Control group (*n* = 16)
Age children, *M* (DT)	5.96 (1.43)	6.19 (1.22)
Girls, *n* (%)	12 (52.2%)	6 (37.5%)
Boys, *n* (%)	11 (47.8%)	10 (62.5%)
Age parents, *M* (DT*)*	40.52 (4.71)	41.00 (5.48)
Mothers, *n* (%)	20 (87.0%)	15 (93.8%)
Fathers, *n* (%)	3 (13.0%)	1 (6.3%)
Number of siblings, *n* (%)		
0	11 (47.8%)	6 (37.5%)
1	10 (43.5%)	6 (37.5%)
2	1 (4.3%)	4 (25.0%)
3	1 (4.3%)	-
Therapy children, *n* (%)		
Yes	5 (21.7%)	-
No	18 (78.3%)	16 (100%)
Cohabitation, *n* (%)		
With both parents, in the same house	19 (82.6%)	14 (87.5%)
With one of their parents and their new partner	1 (4.3%)	1 (6.3%)
With both parents, in alternate houses	2 (8.7%)	1 (6.3%)
Other	1 (4.3%)	-
Marital status, *n* (%)		
Married	19 (82.6)	12 (75.0%)
Cohabitant	1 (4.3%)	2 (12.5%)
Divorced or separated	2 (8.7%)	-
Bachelor	1 (4,3%)	2 (12.5%)
Level of education, *n* (%)		
Higher education	13 (56.5%)	12 (75.0%)
Secondary education or FP	7 (30.4%)	4 (25.0%)
Basic studies	3 (13.0%)	-
Employment status, *n* (%)		
Autonomous	5 (21.7%)	2 (12.5%)
Part-time employee	2 (8.7%)	3 (18.8%)
Full-time employee	11 (47.8%)	9 (56.3%)
Unemployed	4 (17.4%)	2 (12.5%)
Other	1 (4.3%)	-
Income level, *n* (%)		
Between 500 y 999€	5000€ or more	1 (6.3%)
Between 1000 y 1999€	9 (39.1%)	3 (18.8%)
Between 2000 y 2999€	6 (26.1%)	3 (18.8%)
Between 3000 y 4999€	5 (21.7%)	6 (37.5%)
I prefer not to answer	3 (13.0%)	2 (12.5%)
Province of residence, *n* (%)		
Alicante	21 (91.3%)	-
Murcia	2 (8.7%)	7 (43.8%)
Asturias	-	1 (6.3%)
Islas Baleares	-	2 (12.5%)
Las Palmas	-	1 (6.3%)
Madrid	-	4 (25.0%)
Valencia	-	1 (6.3%)

The inclusion criteria were (a) being between 4 and 8 years old and (b) obtaining a score of 32 or higher (50th percentile) on the *Parent Version of the Nighttime Fears Scale* (NFS-P; [26]). Participants were assigned to either the intervention or waitlist control condition using a non-random, quasi-experimental design based on geographic convenience. This approach was chosen to facilitate treatment adherence and logistical feasibility for the families involved. After the 5-week intervention for the experimental group, families in the waitlist control group were offered the opportunity to receive the treatment, with all necessary materials provided for its implementation.

### Instruments

#### Sociodemographic variables

The sociodemographic variables collected for the children included sex, age, number of siblings, household composition, country of birth, province of residence, and whether they were receiving psychological treatment at the time. The sociodemographic variables collected for the parents included sex, age, marital status, education level, employment status, and income level.

#### Parental version of the nighttime fears scale (NFS-P; [26])

The NFS-P assesses the level of nighttime fear in school-age children through their parents. This measure includes four subscales: fear of nighttime features and distressing experiences (items examine stimuli associated with the dark, as a characteristic of the night, which may generate fear), fear of loss or separation from the family (items are related to the loss or absence of attachment figures and other family members), fear of imaginary stimuli (items refer to unreal stimuli, such as ghosts, which may cause fear), and fear of real stimuli (items refer to real stimuli, such as thieves, which may cause fear). The scale consists of 21 items that inquire about nighttime situations likely to induce fear in children. These items are rated on a 5-point Likert scale, ranging from 0 (not at all) to 4 (very much). Higher scores indicate greater intensity of nighttime fears. The internal consistency of the scale is adequate for the overall scale (ordinal *α* = 0.94) and for the subscales (*α* values range from 0.90 to 0.95) [26]. In the present study, the internal consistency showed adequate Cronbach’s alpha coefficients for all subscales: fear of nighttime features and distressing experiences (0.84 pre-test, 0.83 post-test), fear of loss or separation from the family (0.84 pre-test, 0.89 post-test), fear of imaginary stimuli (0.84 pre-test, 0.89 post-test), and fear of real stimuli (0.92 pre-test, 0.91 post-test).

#### Nighttime Behaviors Questionnaire for Children-Parent-reported (NBQC-P; [2])

The NBQC-P evaluates, through parent reports, the behaviors children typically exhibit before going to bed and during the night. It consists of three subscales: need for company to sleep, problems during the night, and problems when going to sleep. This measure includes 13 items, with a 5-point Likert scale ranging from 0 (never or almost never) to 4 (always or almost always). A higher score on each dimension indicates greater need for company to sleep, higher resistance before going to bed, and greater interference from nighttime awakenings. The psychometric properties in the present study were adequate, with lower internal consistency in subscales 2 and 3, possibly due to the small sample size (need for company: *α* pre-test = 0.88, *α* post-test = 0.88; nighttime problems: *α* pre-test = 0.60, *α* post-test = 0.71; problems falling asleep: *α* pre-test = 0.77, *α* post-test = 0.61).

#### What I Can Do at Night-Parent form (WICDAN-P; [6])

This questionnaire aims to assess, through parents, the child’s ability to perform adaptive nighttime behaviors during the past week. The items measure various nighttime behaviors, such as the child’s ability to sleep alone in their room, move around their room in the dark, or stay alone in the dark in their room, among others. The scale consists of 11 items, with a 3-point response scale (0 = no; 1 = yes, with difficulty or indecision; 2 = yes, easily). The total score of the questionnaire ranges from 0 to 22. A higher score indicates more adaptive nighttime behavior. This instrument has adequate psychometric properties (ordinal *α* = 0.91, categorical *ω* = 0.87; [1]). The internal consistency in the present study was adequate, with *α* values of 0.80 (pre-test) and 0.92 (post-test).

In the present study, the English version of the WICDAN-P was translated into Spanish, with prior authorization from the original author of the instrument. The items were translated into Spanish and simplified for better comprehension.

#### Diagnostic interview based on DSM-5 criteria

An individual diagnostic interview was conducted with the parents of each child based on the DSM-5 criteria solely as an inclusion criterion to confirm eligibility; this interview was used to verify whether the children had a phobia of the dark and to rule out other potential psychological issues. Seven questions were asked, with parents responding “Yes” (if the criterion was met) or “No” (if the criterion was not met). The items in the interview were as follows: intense fear or anxiety when in a dark situation (expressed through crying, tantrums, becoming paralyzed, or clinging); darkness almost always triggers immediate fear or anxiety; active avoidance of darkness with intense fear or anxiety; the fear or anxiety is disproportionate to the actual danger posed by the darkness and the sociocultural context; the fear, anxiety, or avoidance of darkness is persistent and typically lasts six or more months; the fear, anxiety, or avoidance causes significant distress or impairment in social or other areas; and the disturbance is not better explained by the symptoms of another mental disorder.

#### Nighttime behavior log

Daily, families completed a log documenting various behaviors of their children during the night. This log included information on the number of minutes it took for the child to go to bed, the number of minutes it took for the child to fall asleep, avoidance behaviors, need for company to fall asleep, presence of a nightlight, number of nighttime calls to the parents’ bed, number of nighttime visits to the parents’ bed, whether the child slept in their own bed the entire night, and, finally, whether the book and/or games were used.

#### Weekly and final semi-structured interviews

Qualitative data were collected through weekly phone calls and a final contact. These semi-structured interviews followed a standardized script designed to monitor the intervention’s progress, ensure the correct use of the book and games, and address any logistical doubts from the families. In the final session, families were also asked about which games were the most enjoyable and most helpful for their children.

### Procedure

**Fig. 1:**
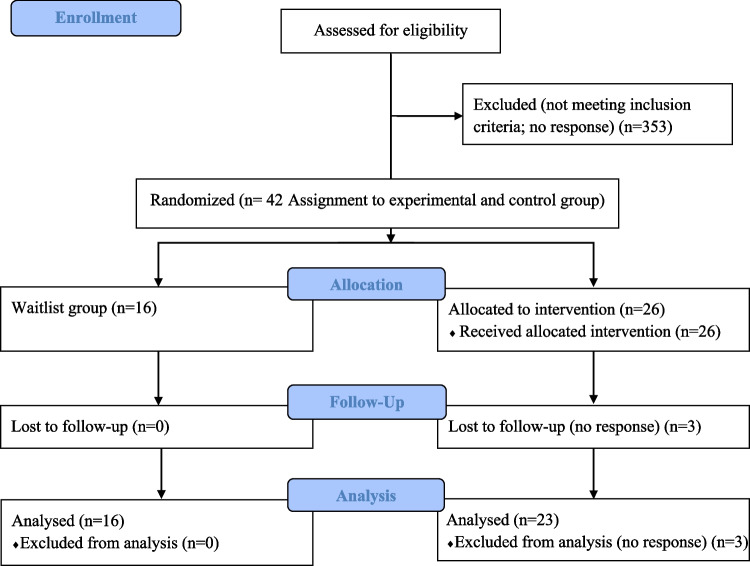
CONSORT 2010 flow diagram of participant recruitment

Participants were recruited between January 2024 and December 2025 through social media platforms such as WhatsApp, Instagram, Facebook, and X (formerly Twitter). The recruitment of participants is shown in Fig. 1. Parents completed an online survey created using the Google Forms platform, which was distributed through a snowball sampling strategy. Prior to answering the questionnaire, parents were informed about the study’s purpose and provided their consent. At the end of the survey, all participants received written guidelines to help their children cope with the fear of the dark. Once the participants for the experimental and waitlist control groups were selected according to the inclusion criteria, the intervention was conducted over a period of 4–5 weeks. After the experimental group completed the program, both groups were re-assessed, and treatment materials were sent to the families in the control group. This study was approved by the Ethics Committee of the Miguel Hernández University and was registered on https://ClinicalTrials.gov (ID: NCT07067320). Data were handled in accordance with the Declaration of Helsinki.

### Treatment

The treatment was based on reading the book *Tío Piesligeros, Enciende la Luz*, the Spanish adaptation of Uncle Lightfoot, Flip that Switch [5]. The home-based intervention combined reading the book (which included 22 chapters) with engaging in various games (18 proposed games to be played). In these games, children were gradually exposed to the feared stimulus (darkness) in a fun way. The games taught coping skills and addressed different common nighttime fears in children, such as nightmares, noises, shadows, and monsters. Other therapeutic components were used, such as positive reinforcement (both material and verbal) and relaxation techniques.

The materials required for the treatment were as follows: (a) *Tío Piesligeros, Enciende la Luz*, the book in which each chapter explained a different game; (b) *Parent Guide*, a document with information for the parents, which included general recommendations on reading and playing the games, as well as detailed instructions on how to play each game; (c) *Nighttime Behavior Log*, to track the children’s behaviors during the night (which included the detailed information mentioned earlier).

An initial session was scheduled with the parents to provide information about fear and the darkness phobia and to explain in detail how to apply the treatment at home. In addition, informed consent was obtained from the families, and each family was given a folder containing all the necessary materials for the treatment. Finally, a diagnostic interview based on the DSM-5 criteria was conducted on an individual basis.

The treatment was applied at home by the parents over 4 or 5 weeks. During this period, weekly phone contact was maintained with the parents to monitor the treatment and resolve any doubts. These calls allowed for qualitative weekly tracking of the children’s progress.

At the end of the treatment, another session was held with the parents to share experiences with the book and games, evaluate the children’s progress qualitatively, and collect the nighttime behavior logs. This session also included the post-test evaluation.

While the term “parents” is used throughout this manuscript to refer to the family unit, it should be noted that in most cases, the intervention was primarily implemented by one single caregiver (predominantly the mother). Both parents were encouraged to collaborate; however, for the purposes of data collection and qualitative interviews, a primary informant was identified for each family to ensure consistency in reporting child progress and treatment adherence.

#### Statistical analysis

Descriptive statistics were calculated using SPSS v25 statistical software for Windows. Frequencies, means, and standard deviations were computed. The analysis of differences in nighttime fears, sleep functioning, and darkness coping skills between pre- and post-intervention measures for the two experimental conditions was conducted using JASP 0.18.1 for Mac. For this, the 2-group × 2-moment repeated measures ANOVA test was performed once the assumptions for its application (normality, homoscedasticity) had been checked. The interaction contrast of the ANOVA test was used. The effect size was calculated using the *ω*^2^ statistic, with the following reference values: < 0.01 = very small, 0.01–0.06 = small, 0.06–0.14 = medium, and ≥ 0.14 = large [12]. This metric was chosen because it provides a less biased estimate of the population variance explained compared to eta squared (*ƞ*^2^), especially in small or moderate-sized samples [24]. A significance level of *α* = 0.05 was used throughout. Non-parametric tests were performed to observe differences between various measures of the nighttime behavior log, using the Wilcoxon test and the McNemar test.

Additionally, qualitative analyses were conducted on participants in the experimental condition. Frequencies were obtained for the games that had been most helpful and enjoyable for the children, as well as for the progress observed by parents in the weekly and final interviews. The progress was grouped into common categories based on content similarity. This process was performed independently by two researchers, and the results were compared. Furthermore, graphs were created with the average values of the variables from the behavior log.

## Results

### Feasibility and treatment adherence

Regarding the feasibility of the intervention, 23 out of 26 families assigned to the experimental group fulfilled the full 5-week program. No missing data were recorded for the participants who completed the post-treatment assessment. A total of 3 families withdrew from the study prior to completion; the primary reasons for early termination were lack of time to implement the games daily and personal family issues. Overall, the high completion rate of 88.5% suggests that the intervention is feasible and well accepted by the target population.

### Differences between groups in age and sex

No statistically significant differences were found in the age of participants (Mann–Whitney *U* = 204, *p* = 0.569) between groups. Likewise, no statistically significant differences were found in sex between groups, *χ*^2^(1) = 0.818, *p* = 0.366.

### Improvements in nighttime fears and functioning

Analyses were conducted using the Student’s *t*-test to examine differences between the experimental and control groups at pre-intervention. No statistically significant differences were found between the groups in the subscales of the NFS-P (fear of nighttime features and distressing experiences, *t* =  − 1.648, *p* = 0.108; fear of loss or separation from the family, *t* =  − 1.369, *p* = 0.179; fear of imaginary stimuli, *t* =  − 1.099, *p* = 0.279; fear of real stimuli, *t* = 0.230, *p* = 0.820). Similarly, no statistically significant differences were found in the subscales of the NBQC-P (need for company to sleep, *t* =  − 1.311, *p* = 0.198; problems during the night, *t* =  − 1.286, *p* = 0.206; problems when going to sleep, *t* =  − 1.841, *p* = 0.074). No statistically significant differences were found in the WICDAN-P test (*t* = 0.827, *p* = 0.413).

Table 2 reflects the differences between the experimental and control conditions between the pre-test and post-test. Regarding the NFS-P, statistically significant time × group interaction effects were found in two out of the four subscales: fear of nighttime features and distressing experiences (*F* = 17.085, *p* < 0.001, *ω*^2^ = 0.078) and fear of imaginary stimuli (*F* = 15.422, *p* < 0.001, *ω*^2^ = 0.066), both showing moderate effect sizes. In contrast, no significant interaction effects were observed for the subscales fear of loss or separation (*F* = 3.116, *p* = 0.086) and fear of real stimuli (*F* = 1.608, *p* = 0.213), indicating that these dimensions did not experience differential changes between groups over time. On the other hand, statistically significant effects were observed in the three factors of the NBQC-P: need for company to sleep (*F* = 11.850, *p* = 0.001, *ω*^2^ = 0.042), problems during the night (*F* = 6.005, *p* = 0.019, *ω*^2^ = 0.028), and problems when going to sleep (*F* = 8.810, p = 0.005, *ω*^2^ = 0.060). Regarding the results obtained in the WICDAN-P instrument, a statistically significant interaction was found, with a large effect size (*F* = 37.147, *p* < 0.001, *ω*^2^ = 0.17).

**Table 2:** Group × time interaction in the dimensions of the NFS-P and NBQC-P between experimental and control groups

	Experimental group		Control group			
	Pre, ***M*** (***DT***)	Post, ***M*** (***DT***)	Pre, ***M*** (***DT***)	Post, ***M*** (***DT***)	***F*** (*p*)	***ω***^2^
**NFS-P**						
Fear of nighttime features and distressing experiences	24.22 (5.931)	15.48 (5.133)	21.13 (5.512)	19.19 (6.145)	**17.085** **(< 0.001)**	0.078
Fear of loss or separation from the family	12.91 (4.252)	9.57 (4.944)	10.88 (5.005)	10.44 (4.926)	3.116 (0.086)	0.016
Fear of imaginary stimuli	10.30 (4.172)	5.61 (3.285)	8.87 (3.722)	8.50 (4.590)	**15.422** **(< 0.001)**	0.066
Fear of real stimuli	8.70 (5.261)	6.91 (4.122)	9.06 (4.328)	9.31 (4.701)	1.608 (0.213)	0.004
**NBQC-P**						
Need for company to sleep	12.17 (4.745)	7.78 (5.744)	10.13 (4.884)	10.19 (4.778)	**11.850 (0.001)**	0.042
Problems during the night	11.13 (3.794)	6.13 (4.352)	8.75 (4.219)	6.88 (4.303)	**6.005 (0.019)**	0.028
Problems when going to sleep	7.30 (4.607)	3.43 (2.727)	5.50 (3.830)	5.62 (3.364)	**8.810 (0.005)**	0.060
**WICDAN-P**_TOTAL	6.13 (4.818)	16.00 (5.469)	7.25 (2.933)	8.13 (5.252)	**37.147** **(< 0.001)**	0.174

Statistically significant interaction effects are indicated in bold.

*M* mean, *SD* standard deviation, *ω*^*2*^effect size.

Qualitatively, various progressions were identified in the weekly and final interviews held with the 23 families. When parents were asked openly about the improvements, they mentioned a decrease in fear and anxiety responses to the dark (*n* = 9; Family 1 [F1]: “I see him more relaxed and calm”; F2: “He is not tense at night”) and greater ability to control these responses (*n* = 5; F3: “He knows when he feels anxious, the story and the protagonist are helping him rationalize things […]. He verbalizes that he has lost his fear of the dark”). They also reported a reduction in resistance to going to bed (*n* = 6; F4: “When we tell him it’s time for bed, he doesn’t take as long as before”) and greater ease in falling asleep (*n* = 4; F5: “He falls asleep very quickly practicing the relaxation techniques he has learned”). The program helped many children learn to sleep alone, without their parents’ company (*n* = 7; F6: “I no longer have to stay for him to fall asleep”) and with less need for light (*n* = 8; F7: “We’ve gone from using a big light to a smaller one”). During the night, families noticed a decrease in calls, visits, and awakenings (*n* = 7; F8: “He doesn’t call us at night”; F9: “He doesn’t get up as often and doesn’t come to our bed”) and improved skills in controlling fear and reactions to nightmares (*n* = 6; F6: “He has fewer nightmares […]. When he has one, he stays calm and doesn’t call us”). They also mentioned that they had learned to move in the dark (*n* = 13; F2: “He can go upstairs when it’s dark”; F8: “Even when he gets up to get water, he goes alone without calling us […]. He doesn’t turn on all the lights”). These progressions have positively impacted the children’s rest (*n* = 3; F10: “She is more rested”) and the families’ rest (*n* = 3; F9: “Now we sleep through the night”).

It is noteworthy that, although parents were explicitly asked about potential difficulties or negative aspects of the program during the weekly and final interviews, no negative reflections or neutral comments regarding the intervention’s efficacy were reported by the participants. All families who completed the study expressed high levels of satisfaction, focusing their feedback on the improvements observed in their children’s nighttime behavior.

### Improvements in nighttime behaviors

**Fig. 2:**
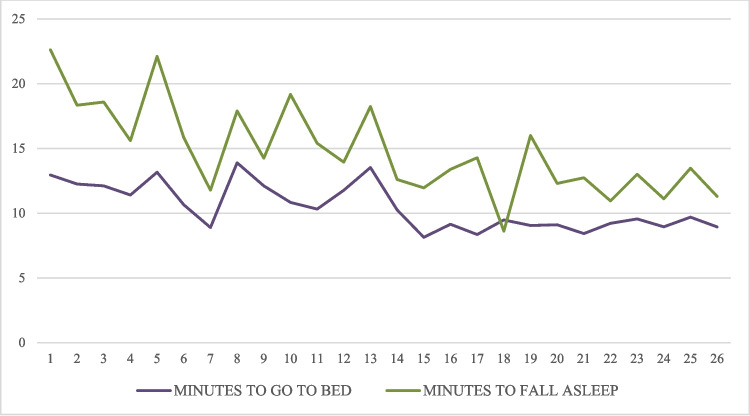
Minutes that children took to go to bed and fall asleep

Figure 2 shows the changes in the records over the course of the intervention in the experimental group regarding the minutes children took to go to bed and fall asleep, from day 1 to day 26 (the last day for which data were available for all children). Despite some fluctuations, the minutes children took to go to bed decreased from an average of 12.95 (*SD* = 15.93) on day 1 to 8.94 (*SD* = 12.20) on day 26. The minutes it took them to fall asleep decreased from 22.62 (*SD* = 16.63) to 11.29 (*SD* = 10.05).

Additionally, Wilcoxon tests were performed to analyze the differences between the first and last days for which data were available for each participant, regarding the variables minutes to go to bed (*Z* =  − 2.722; *p* = 0.006) and minutes to fall asleep (*Z* =  − 3.525; *p* < 0.001). Statistically significant differences were observed in both variables.

**Fig. 3:**
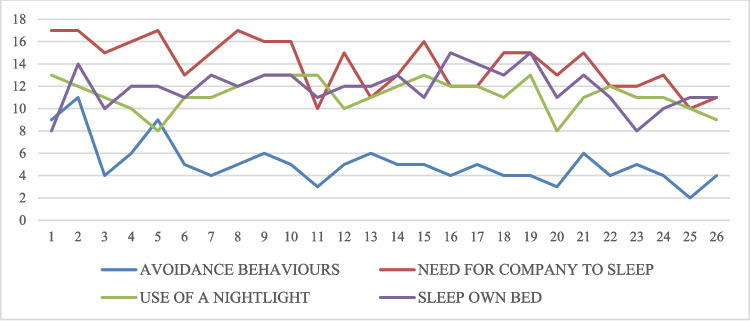
Evolution during the treatment of avoidance behaviors, need for company to sleep, use of a nightlight, and the ability to sleep in their own bed

Figure 3 shows the evolution of the following variables: avoidance behaviors, need for company to sleep, use of a nightlight, and the ability to sleep in their own bed. Although a general descending trend is observed, the data show notable daily fluctuations rather than a linear decrease. In relation to the children’s avoidance behaviors before bed during the weeks of the intervention, on day 1, 9 children displayed these behaviors, while by day 26, 4 children exhibited avoidance behaviors. Regarding the need for company to sleep, it can be observed that on day 1 of the treatment, 17 children needed to be accompanied by their parents to fall asleep, and by day 26, 11 children still showed this need. In relation to the evolution of the need for a nightlight when going to sleep, initially, 13 children required a nightlight, while by day 26, only 9 children still needed it. In terms of the ability to sleep in their own bed throughout the night, on day 1, 8 children showed this behavior, and by day 26, 11 children were able to do so.

Additionally, to determine whether there were statistically significant differences between day 1 and the last day with available data for each participant, the McNemar test was used for the following behaviors: avoidance behaviors (*N* = 3.57; *p* = 0.125), need for company to sleep (*N* = 2.67; *p* = 0.219), use of a nightlight (*N* = 4.50; *p* = 0.070), and the ability to sleep in their own bed (*N* = 8.00; *p* = 0.008). Statistically significant differences were found only in the last variable, ability to sleep in their own bed.

**Fig. 4:**
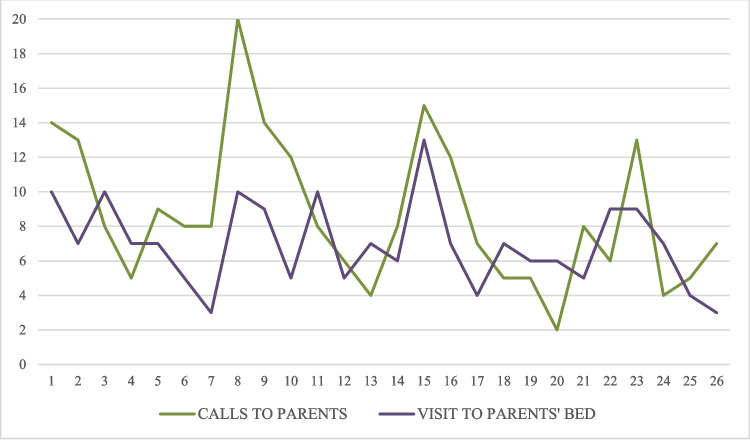
Evolution of the number of calls to parents and visits to the parents’ bed

Figure 4 shows the evolution in the number of calls the children made daily to their parents during the night, as well as the number of visits they made to their parents’ bed. On the first day, a total of 14 calls were made by all the children in the experimental group, while on day 26, 7 calls were made. Similarly, a total of 10 visits were made on the first day (by all children in the experimental group), and a total of 3 visits were made on day 26. While both variables show a general downward trend by day 26, the progression was characterized by high daily instability and significant peaks in symptom frequency.

Finally, Wilcoxon tests were conducted to analyze the differences between the first day and the last day with data for each participant regarding the number of calls to parents (*Z* =  − 1.862; *p* = 0.063) and the number of visits to the parents’ bed (*Z* =  − 1.540; *p* = 0.124), finding no statistically significant differences in either case.

### Use of the book and game evaluation

Regarding the use of the book and/or games, an average of 18.6 days (*SD* = 4.45) was recorded for reading and/or playing the games during the 4–5 weeks of the intervention. The minimum number of days the book was used was 8, and the maximum was 28. In the final session, parents were asked which games had been most helpful to their children and which they had enjoyed the most, with four of them standing out. The game that was most frequently mentioned as the most helpful (*n* = 7) and most enjoyable (*n* = 7) involved finding another person while blindfolded. Other highly enjoyed games included making animal shadows on the wall (*n* = 4) and using muscle relaxation and breathing techniques (*n* = 3).

## Discussion

The aim of the present study was to evaluate the effectiveness of a therapeutic intervention for children with a fear of the dark, based on bibliotherapy and play, implemented by parents at home.

Considering the changes observed in the experimental condition, the program contributed to the reduction of (1) fears related to the inherent characteristics of the night and potential unpleasant experiences, which can be explained by exposure to darkness situations, and (2) fears of imaginary creatures, whose reduction could be attributed to the previous mechanism and the work done in changing the content of nightmares and diverting attention away from them.

Statistically significant reductions were not achieved in the fears related to the loss or separation from family members and in fears of real stimuli associated with a stranger or dangerous person during the night. This lack of significant improvement may be attributed to two main factors. First, the intervention’s components, primarily based on gradual exposure to darkness and cognitive restructuring of imaginary creatures, did not specifically target the social or safety-related anxieties associated with separation or real-world threats (e.g., burglars). Second, developmental factors play a crucial role; while fear of imaginary stimuli typically peaks between ages 4 and 6, concerns regarding physical harm and strangers become more predominant and cognitively complex as children approach ages 7 to 9 [22]. Cognitive restructuring has been shown to increase the effectiveness of behavioral techniques in treating fear of the dark [18], so this component could be useful for addressing fears of this nature in future iterations of the program.

The reduction in nighttime fears is accompanied by an improvement in sleep functioning, in line with the hypothesis proposed and previous studies with bibliotherapy programs [30]. The children who received the intervention showed significantly fewer difficulties in going to bed and falling asleep, as well as an increased ability to sleep in their own bed. During the night, awakenings had less impact on the children’s sleep and their parents’ sleep, as the number of nighttime calls and visits to the parents’ bed decreased. Although these variables showed a slight downward trend in the descriptive analysis, they did not reach statistical significance. This suggests that while the program is effective for reducing core nighttime fears, these specific habitual behaviors may require a longer intervention period or more intensive behavioral reinforcement to be fully extinguished. Future iterations of the program should include components to work more deeply on behavioral advances in order to reduce night-time calls and visits to the parents' bed.

Additionally, requests for accompaniment to fall asleep, bedtime avoidance behaviors, and the need for a security light during the night were reduced. Although these differences were not statistically significant, the improvement found in these variables contributes to better rest, which is relevant considering that persistent sleep difficulties in children are linked to internalizing and externalizing problems, attention-hyperactivity issues, and poor academic performance [19, 20], as well as sleep and mood problems in their family members [31].

The daily records of nighttime behaviors and the considerable standard deviations observed in the pre- and post-test measures reflect a high degree of inter- and intra-individual variability. This daily fluctuation in symptoms suggests that overcoming nighttime fears is not a linear process, but one subject to temporary relapses and environmental influences. Consequently, these periodic oscillations occur before a stable reduction can be achieved at the end of the treatment. Methodologically, the large standard deviations indicate that while the intervention is effective on average, children start from different baseline levels of fear and respond at different paces to bibliotherapy and games.

These improvements in nighttime fears and sleep functioning are related to the acquisition of behaviors during the games. Consistent with previous results [17, 30], the parents of the children who participated in the program—unlike those in the control group—reported improvements in various nighttime behaviors. The participants improved in nighttime skills such as being able to sleep alone all night in their darkened room (with a small light in the hallway or in the room), going to the bathroom alone during the night after everyone else had gone to bed (turning on the necessary lights), or staying alone in bed with the room dark until falling asleep, among others. The improvements in sleep functioning are attributed to the training of these skills through the games that the participants had to engage in after reading the chapters.

The quantitative improvements in skills, functioning, and nighttime fears are qualitatively confirmed by the families’ comments on their children’s progress and the overall evaluation of the program (F3: “For me, this reading has been magic”; F4: “He improved very quickly, almost from the first day”). The progress observed in the children follows patterns found in previous studies [18] on the effectiveness of bibliotherapy with gradual in vivo exposure games to darkness, to which are added modelling with the story’s protagonist, reinforced practice with material and social reinforcement, and the provision of other skills, such as relaxation, which can be useful for initiating or returning to sleep after a nighttime awakening.

Regarding treatment fidelity, the frequency of book usage was monitored through the daily nighttime behavior log, in which parents had to note whether they used the book and games. While the protocol recommended daily reading, the actual usage ranged from 8 to 28 days. This variability in adherence is a common challenge in parent-led home interventions and may have influenced the magnitude of symptom reduction. Specifically, families with lower engagement (closer to 8 days) might have achieved slower stabilization of nighttime fears compared to those with higher fidelity. However, the qualitative feedback suggests that even with irregular usage, the core concepts of the book were integrated into the families’ routines.

Potential cultural factors specific to Spanish children should be considered when interpreting the findings of this study. In this culture, family dynamics often involve later bedtimes and a high degree of physical proximity between parents and children, which may influence the expectations regarding sleep independence. The high levels of parental involvement and the value placed on family closeness in Spain might also explain the strong presence of “Need for company to sleep” observed in our sample, suggesting that interventions should be sensitive to these cultural norms while promoting autonomy.

## Limitations, strengths, and future research directions

Despite the positive results obtained, several limitations can be mentioned in this study. First, the use of a non-random, quasi-experimental design based on geographic convenience introduces potential selection bias that must be addressed. This assignment method poses certain threats to internal validity, as uncontrolled extrinsic factors (such as socioeconomic status or parenting styles) may have overlapped with the intervention effects. Consequently, these factors limit the strength of our causal inferences. Future research should employ randomized controlled trials (RCTs) to mitigate these selection biases and more robustly establish the causal link between bibliotherapy-based play and the reduction of nighttime fears.

Another limitation of the study is the exclusive reliance on parental reports for assessing the child’s fears and behaviors. By not including the child’s perspective, this study may have missed some valuable information. Future research should consider a multi-informant approach. Due to the children’s age, the measures used were reported by the parents, which means the responses were based on the children’s observable behaviors and verbalizations.

Regarding treatment fidelity, although monitored through daily nighttime behavior logs, not all families followed the program uniformly, and in some cases, obstacles arose during the intervention that affected its consistent implementation, suggesting that family adherence may be related to the intervention’s outcomes.

However, this last limitation is, at the same time, a strength, as the intervention demonstrates favorable changes when applied flexibly and adapted to the circumstances of each family and the difficulties of each child. Additionally, it shows the utility of a novel implementation framework that combines different aspects already present in other bibliotherapy programs [10, 15, 17, 30]. In this case, well-established techniques are also useful when the book is read only once on consecutive nights—within the possibilities of each family—with an initial and final meeting and a weekly call (minimal contact with the therapist). Periodic contact with the families facilitates the implementation of corrective actions. However, it is the families who are responsible for the progress, which demonstrates their importance in the intervention. Finally, following the recommendations from the review by Lewis et al. [18], the design incorporates the evaluation of compliance with DSM-5 criteria for darkness phobia. This action allows for the delimitation of the problem, understanding its causes, and ensuring the clinical utility of the treatment.

In future studies, it would be interesting to monitor the fidelity of the implementation and its relationship with therapeutic change. It would also be important to study those games with greater efficacy in each case, considering possible comorbidities. Additionally, for the evaluation of effects, it would be advisable to use objective measures, such as actigraphy, to monitor the child’s sleep patterns [18], as well as include interviews with the children themselves in order to contrast their perceptions with those of their families.

## Conclusions

The bibliotherapy intervention based on play, applied by parents at home, has generated positive results in overcoming fear of the dark in the children who participated. A reduction in fears related to the characteristics of the night and imaginary stimuli was observed. The need for company to fall asleep has decreased, as well as resistance to going to bed (with a decrease in time taken to go to bed and fall asleep) and difficulties during the night related to awakenings. All of this is associated with the improvement of skills to stay and move in dark environments, especially in the children’s room. These results confirm that bibliotherapy combined with gradual in vivo exposure games and other techniques is an effective intervention method for children aged 4 to 8 with fear of the dark. This treatment modality, applied by parents, is efficient, requires minimal contact with the therapist, and can be adapted to the child’s natural characteristics and context.

## Data Availability

The datasets generated and analyzed during the current study are not publicly available in order to protect participant anonymity.
